# Evidence that the endosomal sorting complex required for transport-II (ESCRT-II) is required for efficient human immunodeficiency virus-1 (HIV-1) production

**DOI:** 10.1186/s12977-015-0197-x

**Published:** 2015-08-14

**Authors:** Bo Meng, Natasha C Y Ip, Liam J Prestwood, Truus E M Abbink, Andrew M L Lever

**Affiliations:** Department of Medicine, University of Cambridge, Addenbrooke’s Hospital, Cambridge, UK; Centre for Childhood White Matter Disorders, VU University Medical Centre, Amsterdam, The Netherlands

**Keywords:** HIV, ESCRT, Late domain, Virus budding

## Abstract

**Background:**

Egress of a number of different virus species from infected cells depends on proteins of the endosomal sorting complexes required for transport (ESCRT) pathway. HIV has also hijacked this system to bud viruses outward from the cell surface. How ESCRT-I activates ESCRT-III in this process remains unclear with conflicting published evidence for the requirement of ESCRT-II which fulfils this role in other systems. We investigated the role of ESCRT-II using knockdown mediated by siRNA and shRNA, mutants which prevent ESCRT-I/ESCRT-II interaction and a CRISPR/Cas9 EAP45 knockout cell line.

**Results:**

Depletion or elimination of ESCRT-II components from an HIV infected cell produces two distinct effects. The overall production of HIV-1 Gag is reduced leading to a diminished amount of intracellular virion protein. In addition depletion of ESCRT-II produces an effect similar to that seen when ESCRT-I and -III components are depleted, that of a delayed Gag p26 to p24 +p2 cleavage associated with a reduction in export of virion particles and a visible reduction in budding efficiency in virus producing cells. Mutants that interfere with ESCRT-I interacting with ESCRT-II similarly reduce virus export. The export defect is independent of the decrease in overall Gag production. Using a mutant virus which cannot use the ALIX mediated export pathway exacerbates the decrease in virus export seen when ESCRT-II is depleted. ESCRT-II knockdown does not lead to complete elimination of virus release suggesting that the late domain role of ESCRT-II is required for optimal efficiency of viral budding but that there are additional pathways that the virus can employ to facilitate this.

**Conclusion:**

ESCRT-II contributes to efficient HIV virion production and export by more than one pathway; both by a transcriptional or post transcriptional mechanism and also by facilitating efficient virus export from the cell through interactions with other ESCRT components.

**Electronic supplementary material:**

The online version of this article (doi:10.1186/s12977-015-0197-x) contains supplementary material, which is available to authorized users.

## Background

Budding of human immunodeficiency virus-1 (HIV-1) from an infected cell is known to be mediated by the endosomal sorting complexes required for transport (ESCRT) machinery recruited by either TSG101/Vps23 (human protein and yeast homologue, respectively) of ESCRT-I and/or ALIX, both via the p6 domain of Gag, the major structural protein of the core of HIV [1]. The amino acid motifs in p6 critical for these two pathways are PTAP and YPXnL (where X refers any amino acid and n = 1–3 residues; abbreviated to YPXL hereafter), respectively. Both pathways require the downstream ESCRT-III and VPS4 ATPase. ALIX is able to recruit CHMP4 of ESCRT-III directly, however its contribution to HIV-1 budding appears to be minimal in certain cell lines unless it is overexpressed [2–4]. For the pathway mediated by TSG101, the link between ESCRT-I and -III is unclear. In yeast, ESCRT-I, -II and -III act consecutively in the formation of multivesicular bodies and ESCRT-II is crucial for the activation of ESCRT-III [5].

ESCRT-II is a Y-shaped complex comprised of two EAP20/Vps25 subunits, each forming one arm, and one copy each of EAP30/Vps22 and EAP45/Vps36 which together form the third arm [6–8]. ESCRT-II binds to ubiquitinated cargos and to membranes [7, 9, 10]. Together with ESCRT-I, it deforms membranes and induces bud formation in vitro [11]. Both copies of the ESCRT-II subunit EAP20/Vps25 are required to induce conformational changes and to activate ESCRT-III CHMP6/Vps20 for membrane scission [8, 12, 13].

In the context of HIV-1 budding, previous reports showed that siRNA mediated depletion of the ESCRT-II subunit EAP20 did not appear to affect virus or virus-like particle (VLP) release [14, 15] and it was concluded that the infectivity of viruses was unaffected up to 48 h after knockdown [14]. Effects at later time points were not investigated in detail although knockdown of CHMP6 reduced infectious HIV-1 production four to five fold at 48 h; this however was ascribed to a general effect on protein trafficking. More recently, using a giant unilamellar vesicular system and full length myristylated HIV-1 Gag, it was shown that whereas ALIX can directly recruit the key ESCRT-III subunit CHMP4, ESCRT-I can only recruit CHMP4 when ESCRT-II and CHMP6 are present as intermediary factors [16]. ESCRT-II was also believed to be dispensable for normal cytokinesis [17] but a more recent report shows that EAP20/Vps25 is required for the recruitment of CHMP6 for ESCRT-mediated abscission [18].

Following an initial observation that ESCRT-II knockdown appeared to inhibit HIV-1 production we analysed its role in viral replication using a range of assays including: knockdown with interfering RNA, dominant negative protein expression and the use of an EAP45 knockout cell line. We present evidence consistent with ESCRT-II having at least two roles in efficient HIV-1 production, one of which is enhancement of viral budding.

## Results

### Knockdown of ESCRT-II with shRNA reduces the production of infectious virus

**Fig. 1:**
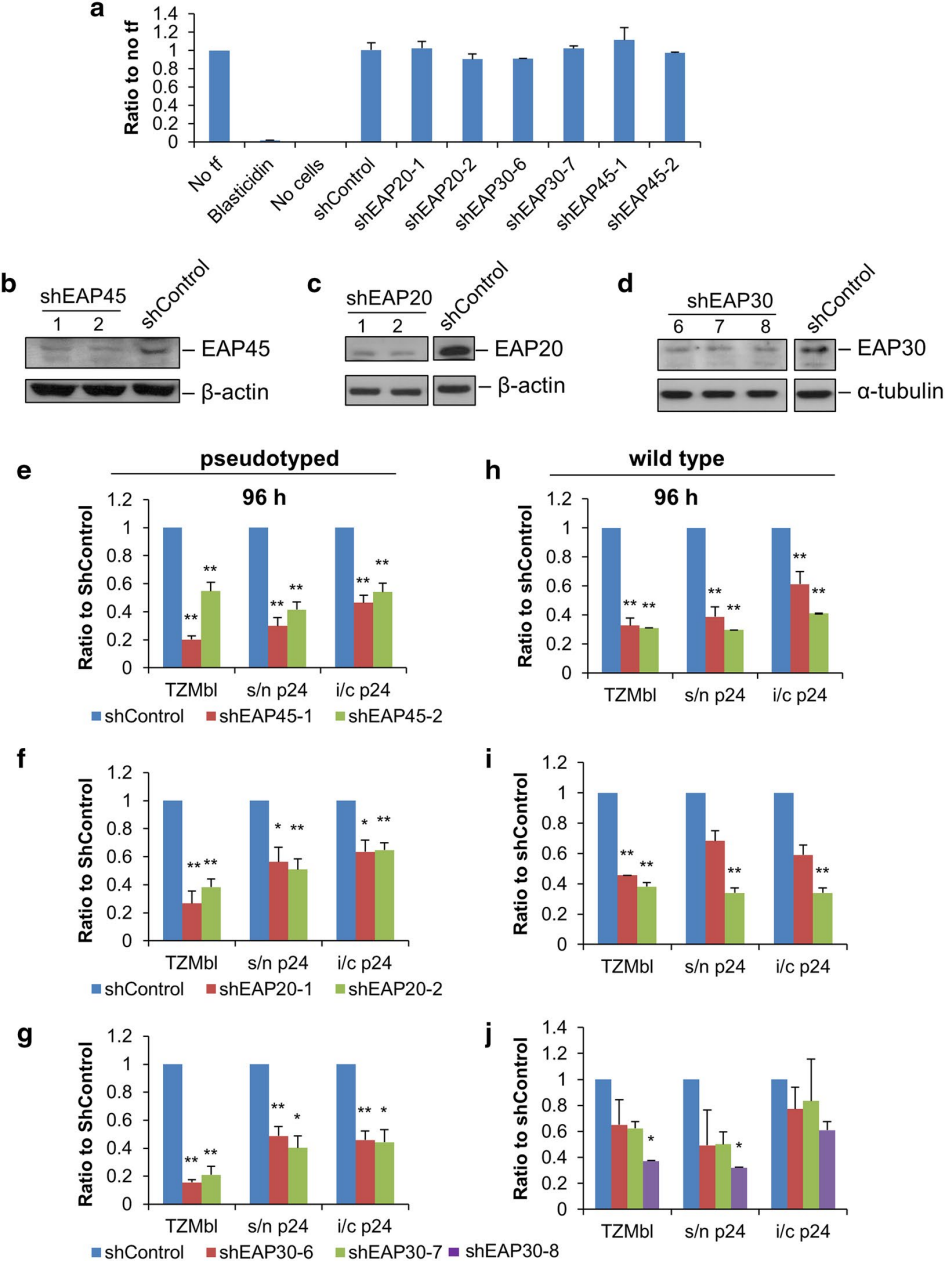
Inhibition of infectious virus production by knockdown of ESCRT-II. **a** Cell viability upon knockdown of ESCRT-II subunits was determined using CellTiter-Glo Luminescent Cell Viability Assay (Promega, n = 2) and normalised to that of the non-transfected control (no-tf). Cells treated with blasticidin served as a control for the viability assay. **b**–**d** Knockdown of ESCRT-II subunits in HeLaM cells. Cells were transfected with 50 ng shRNA expression plasmids using Fugene HD (Roche). Cells were harvested 96 h post-transfection and the levels of EAP20, EAP30 and EAP45 were analysed by Western blots. Detection of β-actin and α-tubulin showed equal loading. **e**–**j** Production of infectious pseudotyped (**e**–**g**) and wild type viruses (**h**–**j**) upon knockdown of ESCRT-II subunits: EAP45 (**e**, **h**), EAP20 (**f**, **i**) and EAP30 (**g**, **j**). The levels of supernatant (s/n p24) and intracellular (i/c p24) CA-p24 were quantified using ELISA. Virus infectivity was determined by infecting TZMbl cells with equal volumes of supernatant from the virus-producing HeLaM cells. Pseudotyped virus (**e**–**g**) was harvested at 96 h post-transfection (n ≥ 3). Wild type virus (**h**–**j**) was harvested at 96 h post-transfection (n ≥ 2). All bars represent the standard error of the mean (SEM); n = number of independent experiments. Unpaired, two-tailed Student’s t test with unequal variance was performed. In all figures *p < 0.05; **p < 0.01; ***p < 0.001.

To minimise confounding off-target effects from individual short hairpin RNAs (shRNA) and to serve as independent controls for the same target, up to three shRNA expression plasmids were constructed targeting the individual subunits of ESCRT-II (Table 1). They were transiently transfected into cells. We confirmed by Western blot at 96 h post transfection that cell viability was not affected by the knockdowns (Fig. 1a) and that the targeted subunits of ESCRT-II were effectively depleted (Fig. 1b, c, d). In agreement with previous publications, we also noted that the expression of ESCRT-II proteins was mutually dependent; knockdown of one ESCRT-II subunit led to a reduction of all three (data not shown) [19, 20]. We validated the shRNA system for the study of virus replication using DDX3 as a positive control (Additional file 1: Figure S1) [21]. To assess ESCRT-II in the context of a single cycle of virus replication we cotransfected into cells a combination of plasmids capable of producing vesicular stomatitis virus-glycoprotein (VSV-G)-pseudotyped HIV-1 together with shRNA expression vectors targeting one each of the ESCRT-II components. We measured production of HIV Gag p24 capsid (CA) protein and its release using a CA-p24 ELISA. The infectivity of the pseudotyped particles generated from cells was analysed by TZMbl infectivity assay. By 48 h there was evidence of inhibition of both p24 production and virion infectivity (Additional file 2: Figure S2). Following a media change at 72 h this was more readily seen at 96 h by which time all the shRNAs that had effectively knocked down their targets also caused significant inhibition of viral replication (Fig. 1e, f, g).

**Table 1:** Sequences targeted by the shRNA expression plasmids used

shRNA	Target sequence
shControl	TGGTTTACATGTCGACTAA
shEAP20-1	CGTCAAGCTACAGCGAAAG
shEAP20-2	CCTCGAGTGGTTGGATAAG
shEAP30-6	GGAACTACATCAACAGGTG
shEAP30-7	CCAGGATGTCAGTCAAGAT
shEAP30-8	ACCTGATCAGAGCCATCAA
shEAP45-1	GAATAAGGGCTGTAGGAAT
shEAP45-2	TGATCAAGGCTAAGGAAAT

The target sequence for shControl was derived from Dharmacon ON-TARGET*plus* siRNA. The remaining were selected using Clontech RNAi Target Sequence Selector (http://bioinfo.clontech.com/rnaidesigner/sirnaSequenceDesignInit.do) and MWG Biotech websites (http://www.eurofinsdna.com/products-services/sirna/sirna-design.html).

To ensure that this was not an artefact of the VSV-pseudotype system the experiment was repeated using wild type HIV. Virus production was again analysed 96 h post-transfection and similar results were obtained (Fig. 1h, i, j). Knock down of individual ESCRT-II components thus impairs HIV-1 protein production. There is a decrease in intracellular p24 protein detected but, most markedly by 96 h, we also noted a relatively greater decline in supernatant p24 and viral infectivity compared to the fall in intracellular p24 suggestive of an additional budding defective phenotype.

### Disrupting ESCRT I/ESCRT-II interaction inhibits production of infectious virus

**Fig. 2:**
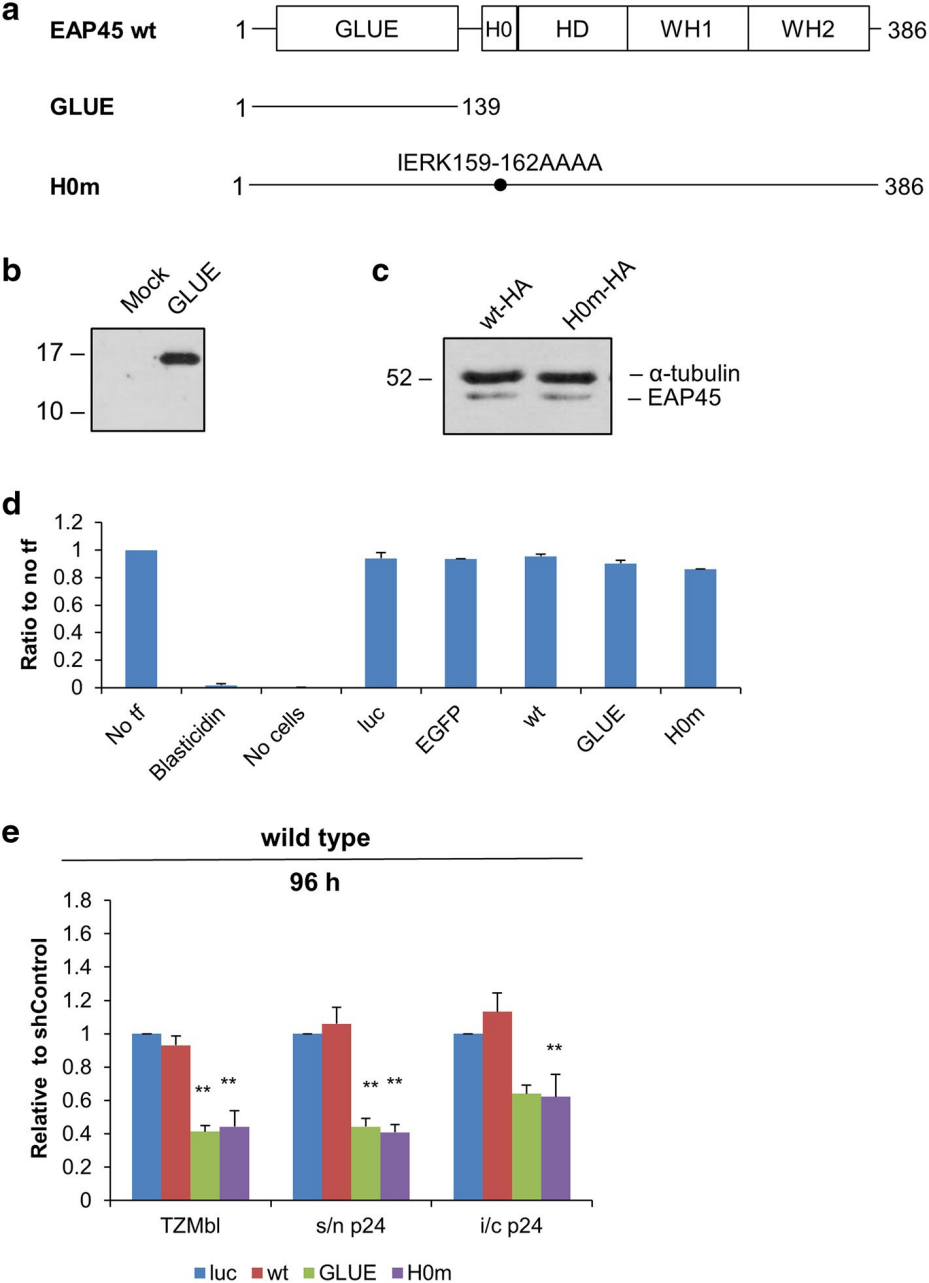
Reduction in infectious virus production by EAP45 mutants. **a** Domain organisation of EAP45 and mutants used in this study. **b** Western blot detecting the expression of GLUE as a 16 kDa protein by a polyclonal rabbit anti-EAP45 antibody. **c** HA-tagged wt or H0M EAP45 expression plasmids were transfected into the cells. The level of expression was visualised by Western blot using anti-HA antibody. Tubulin is served as a loading control. **d** Cell viability assay performed as described for Fig. 1a. **e** Productions of infectious wild type viruses upon co-transfection of EAP45 mutants harvested 96 h post-transfection are shown. Transfections and analyses were performed as in Fig. 1. *Error bars* representing the SEM from up to six replicates are shown.

The EAP45 component of human ESCRT-II contains a GLUE (GRAM-like ubiquitin-binding in EAP45) domain followed by the linker H0 helix, a helical domain (HD) and two winged helix (WH) domains (Fig. 2a) [7]. EAP45 binds to ubiquitin via its GLUE domain [22]. Together with EAP30, the EAP45 GLUE domain also targets ESCRT-II to endosomal and non-endosomal membranes. Moreover, the H0 helix plays an important role for ESCRT-II binding to VPS28 of ESCRT-I. A four amino acid mutation in H0 (H0m) substantially reduced the interaction in vitro [7]. An isolated GLUE domain was also not sufficient to interact with ESCRT-I [7].

We used two mutants (an isolated GLUE domain and the EAP45 mutant IERK159-162AAAA, designated H0m below) to seek evidence of a direct interaction occurring between ESCRT-I and ESCRT-II in HIV particle budding. As determined by Western blot, transfection of GLUE alone led to overexpression (Fig. 2b). H0m transfected and untransfected cells showed no difference in EAP45 expression levels due to interdependency of the ESCRT-II subunits (data not shown). The untagged H0m could not be distinguished from wild type EAP45 on Western blot due to the few amino acid changes, but an HA-tagged variant verified that the mutations in H0m did not prevent its expression (Fig. 2c). The transfection of either mutant did not affect cell survival (Fig. 2d).

Viral plasmids were co-expressed with either wild type EAP45 or one of the two mutant EAP45 proteins. Compared to cells transfected with the control firefly luciferase, wild type EAP45 had no effect on the production of infectious wild type virus (Fig. 2e). By contrast, overexpression of GLUE or expression of H0m, significantly and reproducibly resulted in a decline in intracellular p24 but a proportionally larger fall in extracellular p24 and virion infectivity at 96 h post-transfection (Fig. 2e). Since the GLUE domain is the cargo-binding domain of ESCRT-II and both GLUE and H0m interact less efficiently with ESCRT-I than with EAP45 [7], their expression in HIV-1-producing cells likely diverted cargos that are relevant to viral particle production from the ESCRT machinery. Thus a direct interaction between ESCRT-I and ESCRT-II would appear to facilitate the normal production of infectious HIV-1.

### Knockdown of either TSG101 or EAP20 produces a similar phenotype

**Fig. 3:**
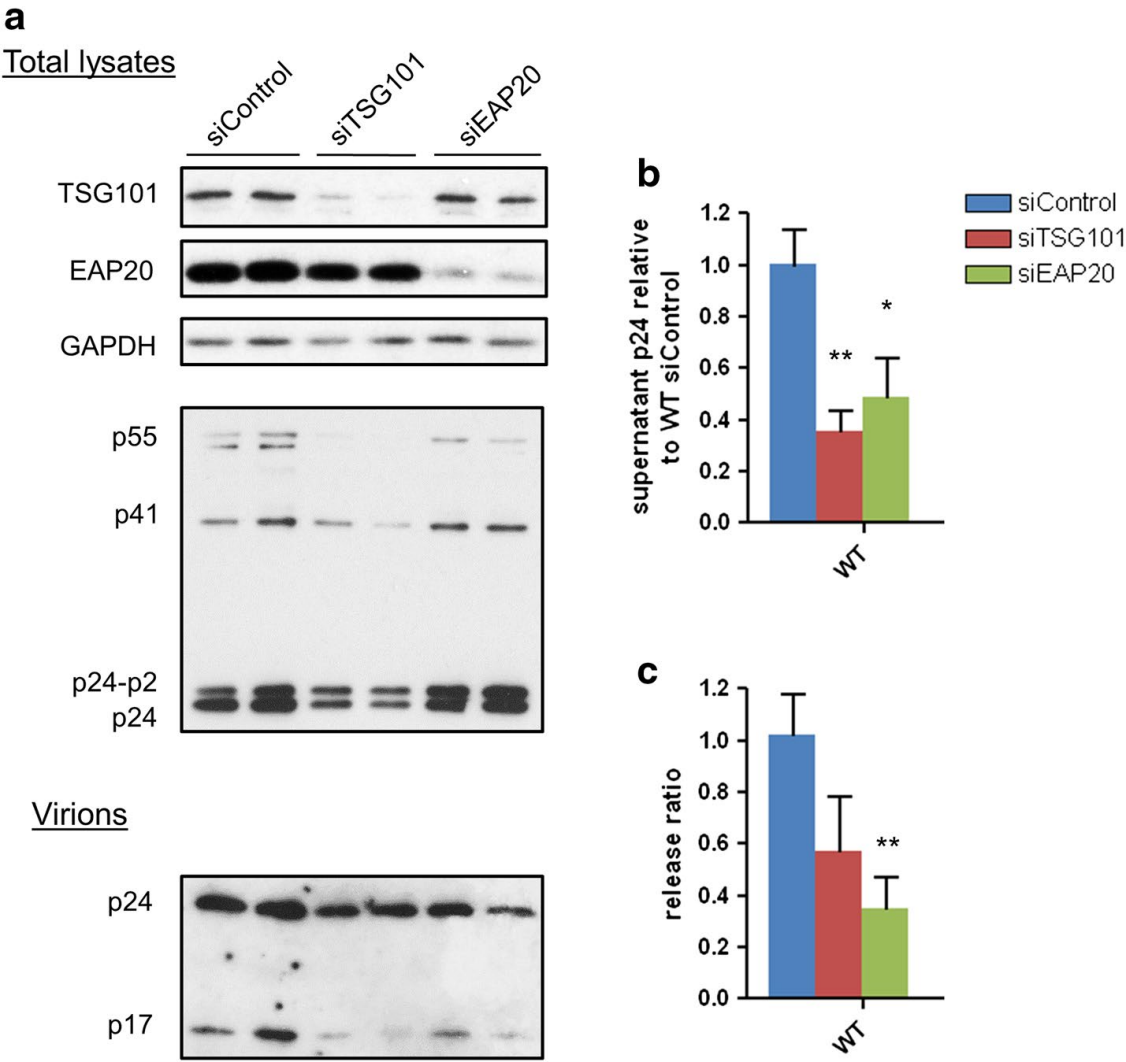
EAP20 is involved in virus release. **a** 293T cells were transfected with siControl, siTSG101 or siEAP20. Plasmids generating HIV pseudotyped with VSV-G were co-transfected with the second dose of siRNA post 24 h transfection. 48 h post second transfection total cell lysates were harvested and the supernatant was cushion-purified followed by Western blot analyses. **b** The supernatant extracellular p24 from WT transfected cells under each condition was normalised to that of siControl. **c** Extracellular p24 is divided by intracellular p24 to give the release ratio before being normalised to siControl. *Error bars* represent the SEM from six replicates. Statistical analyses were done using two-tailed Student t test with the statistical significance shown by *asterisks*.

We further investigated the involvement of ESCRT-II in HIV budding using siRNA targeted against EAP20 and compared it to disrupting ESCRT-I. As expected knockdown of TSG101 led to a late domain defective phenotype with reduced virus export and impaired terminal cleavage of p24/p2 to p24 (Fig. 3a). This was shown by Western blot and confirmed by image analysis (data not shown) and ELISA (Fig. 3b, c) and the measurements converted into a ‘release ratio’. Strikingly the same phenotype of Gag processing and virus release was evident in the EAP20 knockdown cells, again supporting a role for ESCRT-II in HIV budding from the cell in concert with ESCRT-I.

### Knockdown of ESCRT-II has an additive effect on release of YPXL mutant virus

**Fig. 4:**
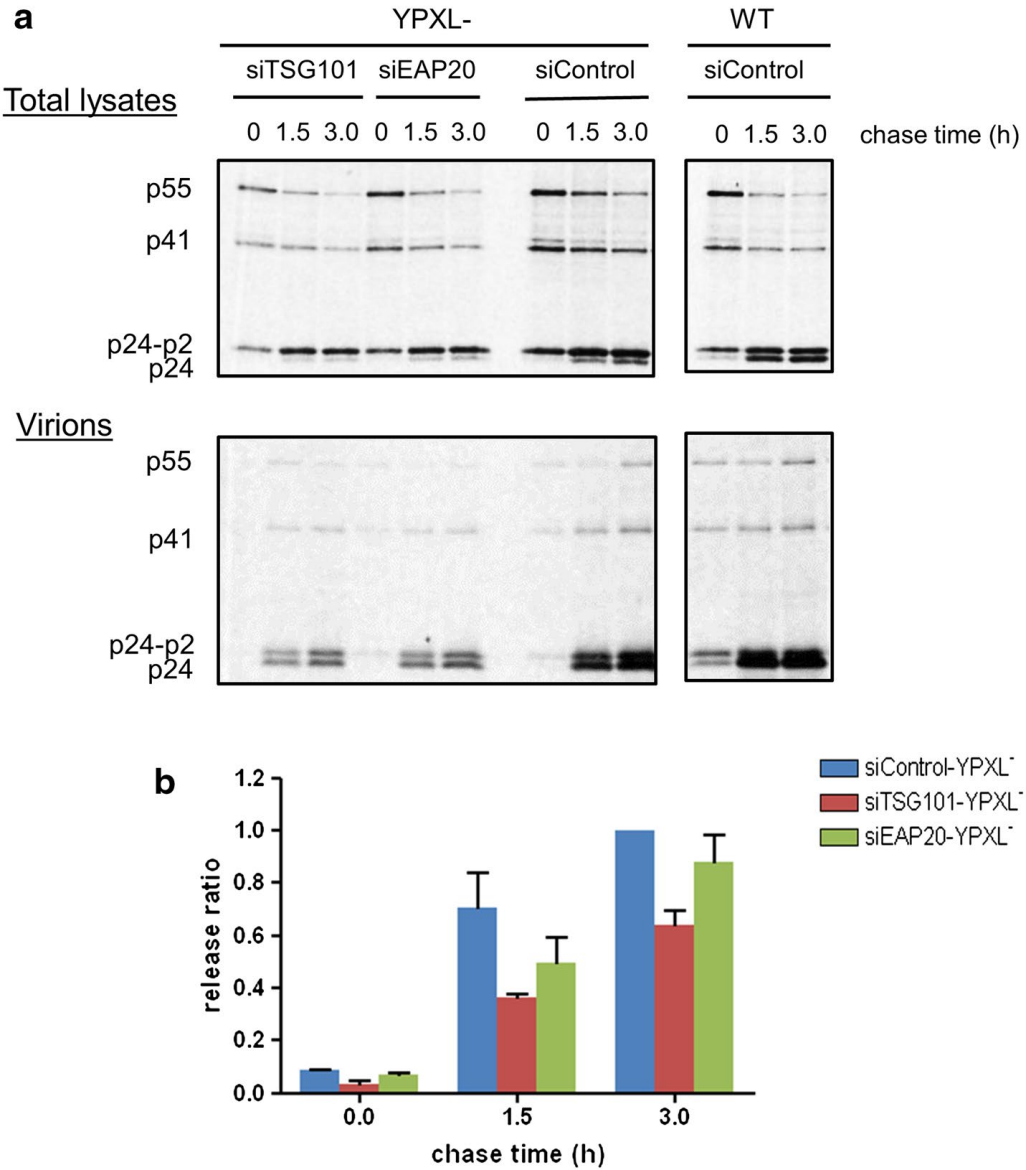
Pulse-chase experiment on YPXL mutant and WT viruses showing the virus production is affected after TSG101 or EAP20 is knocked down. **a** Total cellular lysates and supernatants were harvested at time points 0, 1.5 and 3 h post pulsing under siTSG101, siEAP20 or siControl conditions followed by IP and SDS-PAGE using p24 specific antibody. The predicted viral protein sizes are noted at the *left*. **b** The release ratio was defined as the viral protein in the virions divided by that in the total cell lysates. The ratio at each time point was then normalised against that of YPXL mutant under siControl conditions at 3 h. The *error bars* represent the SEM and were calculated based on three replicates.

If inhibiting viral export by reducing ESCRT-II is occurring through interference with the PTAP pathway, as occurs with TSG101 knockdown, then blocking the alternative ALIX pathway should produce an additive effect when ESCRT-II is depleted. To investigate this possibility we used a Gag protein mutated in the YPXL motif [27]. This mutation of YPXL does not affect viral gene expression (Additional file 3: Figure S3A). We observed a reduction in virus release upon EAP20 knock down similar to that seen when TSG101 is depleted (Additional file 3: Figure S3B, C). We followed the effects on the release ratio in ‘real time’ by performing pulse chase experiments. The YPXL mutation shows reduced virion export which is further exacerbated when either TSG101 or EAP20 is depleted by siRNA reduction, compared to WT (Fig. 4), consistent with the previous observations. The degree of perturbation detected is modest but this likely relates to the relatively fast kinetics of virion assembly relative to the time points of the chase.

### Virus production is affected in CRISPR/Cas 9 EAP45 knockout cells

**Fig. 5:**
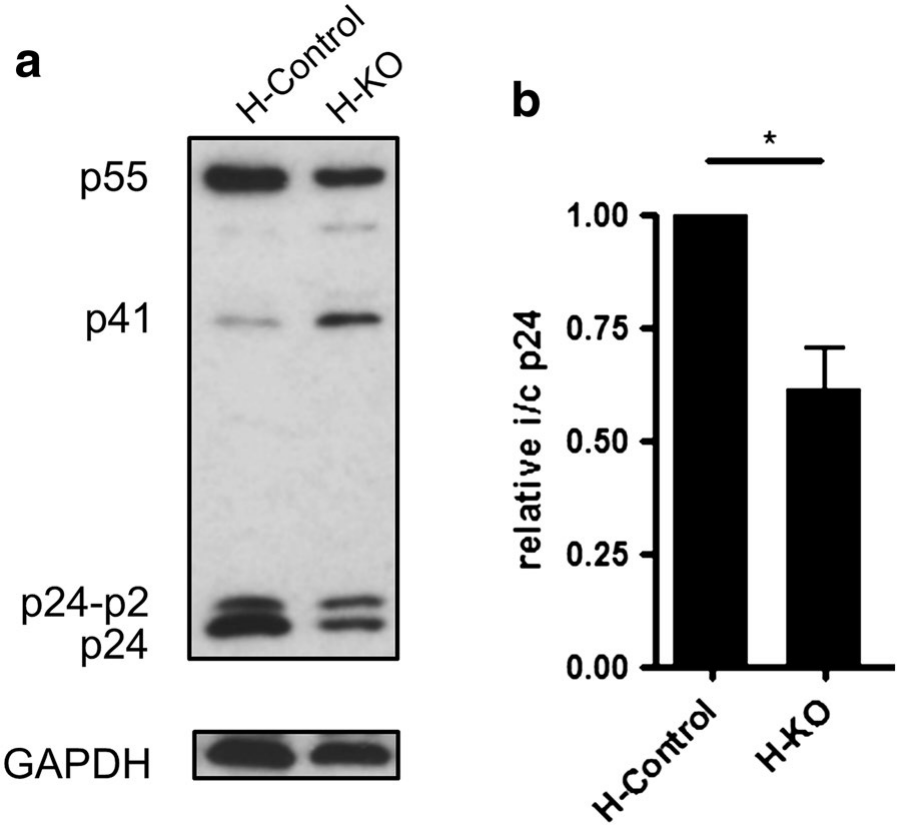
Intracellular Gag is reduced in HAP1 KO cell line. **a** Cells were transfected with plasmids able to produce pseudotyped HIV. 48 h after transfection, the supernatant was removed and cells were lysed for Western blot analyses. GAPDH was a loading control. Viral Gag products are labelled at the *left*. **b** Quantitation of Western blots shown in **a** using Image J. The total p24-associated viral products were quantified and the sum normalised to that of GAPDH preceded by normalisation to WT. *Error bar* represents SEM from five replicates and statistics were done by using a paired two-tailed Student t test; statistical significance is shown by the *asterisk*.

**Fig. 6:**
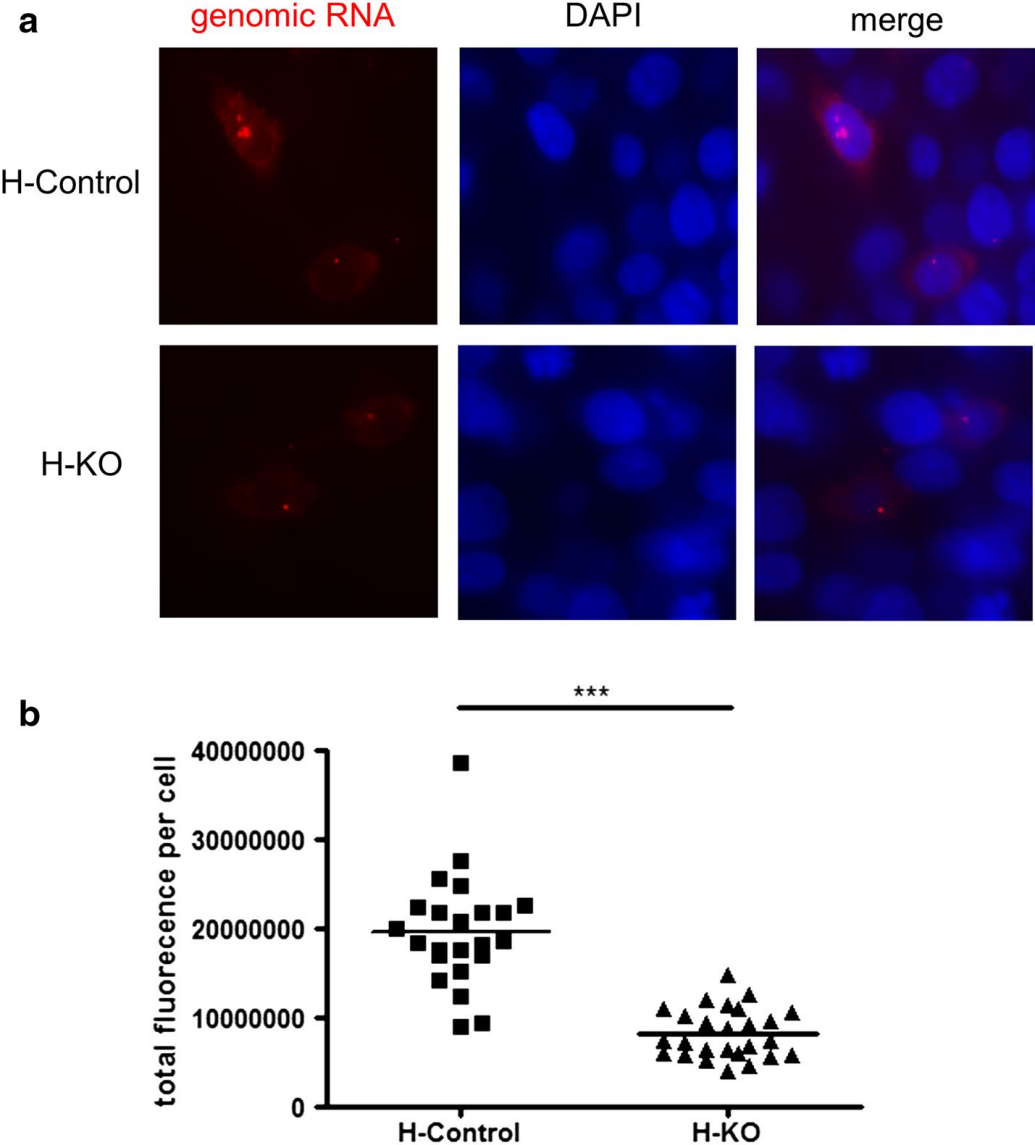
RNA FISH analyses of HIV infected HAP1 cells. **a** The same number of either HAP1 control cells or HAP1 knockout cells was infected with pseudotyped HIV. 24 h after infection, the supernatant was removed and the cells subjected to RNA FISH analysis by fluorescence microscopy. **b** The area of each individual genomic RNA positive control or knockout cell was defined and the fluorescence intensity was quantitated using image J. A total number of 23 or 26 cells, respectively, was identified from either control cells or knockout cells in two experiments. The statistics were done using unpaired two-tailed Student t test with the statistical significance shown by *asterisks*.

Interfering RNA experiments have potential drawbacks in that there may be incomplete knockdown of the target protein and that only very low levels of the factor targeted may be sufficient to carry out the process being probed. We sought to eliminate these concerns using a CRISPR/Cas 9 knock out EAP45 HAP1 cell line constructed by targeting exon 3 of the EAP45 gene (NM_016075). Sanger sequencing confirmed the 20 nt deletion within exon 3 (Additional file 4: Figure S4A). Parental and EAP45 knockout (KO) HAP1 cells were seeded in a 24 well plate and cells were transfected with pseudotyped HIV. The transfection efficiency is comparable as evidenced by the similar level of GFP expressing cells (Additional file 4: Figure S4B). The viability of KO cells is similar with that of parental cells suggesting that absence of EAP45 does not cause deleterious effects on cell proliferation (Additional file 4: Figure S4C). 48 h post transfection, cells were lysed and supernatant harvested for virion purification. Western blot demonstrates a decrease in p24 associated viral products from the EAP45 knockout cell line compared to the parental cell control (Fig. 5). This result is consistent with the shRNA knockdown experiment where a reduction in intracellular p24 was also observed (Fig. 1). To further investigate at what stage this occurs, the HAP1 cells were infected with equivalent amounts of pseudotyped HIV-1 vector and the amount of HIV specific RNA transcripts was measured 24 h post infection by visualisation under fluorescence microscopy using Quasar^®^ 570 Dye labelled multiple oligonucleotide probes (Fig. 6a). The fluorescence intensity at 300 ms exposure time was measured. The level of HIV RNA is noticeably decreased in the HAP1 knockout cell line in comparison with that of the control cell line (Fig. 6b). This would be consistent with a defect in transcription caused by EAP45 knockout. A decline in Gag protein causing a reduced stability of genomic RNA would be unlikely to account for the reduced detection of RNA seen as much of the signal is nuclear and Gag/genomic RNA interactions in HIV are not thought to occur within the nucleus.

A role for EAP45 solely at a transcriptional or early post transcriptional stage would not explain the disproportionate decrease in virion release from ESCRT-II knocked down cells compared to the intracellular levels of viral p24 protein (Fig. 1). In addition the rate of Gag processing appeared to be impaired in the EAP45 knockout cells, especially the terminal cleavage of p24/p2 to p24 compared to the parental cells (Fig. 5).

**Fig. 7:**
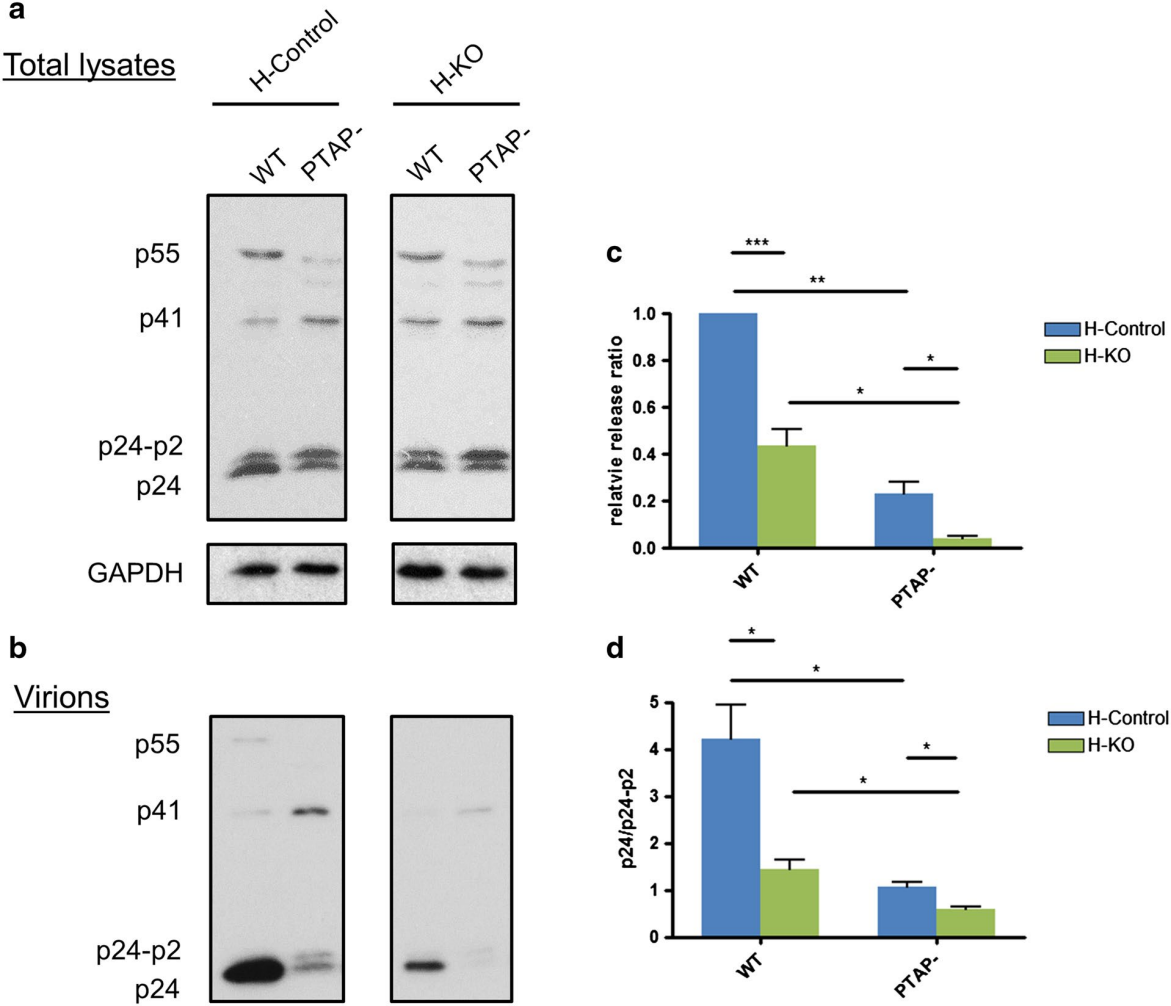
HIV budding is affected in EAP45 knockout HAP1 cells. **a**, **b** Both control and EAP45 knockout cells were transfected with either WT or PTAP deleted mutant (PTAP^−^) pesudotyped HIV. 48 h after transfection, the supernatant was collected for virion purification (**b**) and cells were lysed (**a**) for Western blot analysis. GAPDH was used as a loading control. The viral Gag products are labelled at the *left*. **c** The viral protein detected in **a** was quantified using Image J (NIH) and the release ratio was defined by the ratio of p24 associated viral products in virions divided by that in the corresponding cell lysate, normalised to the level of GAPDH. **d** The p24 and p24-p2 from either WT or PTAP^−^ provirus transfected cells shown in **a** were quantified using Image J. The p24/p24-p2 processing rate is shown by calculating the comparative intensity of p24 and p24-p2. *Error bars* represent the SEM from four replicates. Statistical analyses were done using paired two-tailed Student t test with the statistical significance shown by *asterisks*.

To explore this further we compared virus export in the HAP1 EAP45 knockout and HAP1 wild type cells. Identical numbers of each cell type were transfected with pseudotyped HIV expressing either wild type Gag or PTAP mutated Gag. 48 h after transfection, the supernatant was collected and virions purified by ultracentrifugation; cells were lysed for Western blot analysis. As expected, the PTAP mutant produced less virions from both cell types (Fig. 7a). However in addition we noted a disproportionate decrease in virus export from the EAP45 knockout cells (Fig. 7b). Quantitation of the release ratio after normalisation to the total p24-associated viral products shows that there is a significant reduction in virion release from EAP45 knockout cells (Fig. 7c). This suggests that while ESCRT-II is not absolutely required for budding its presence promotes efficient virion release. Knock out of EAP45 produces a defect in the final cleavage of p24 from p24-p2 (Fig. 7d) substantiating the involvement of ESCRT-II at the final stage of virus budding.

**Fig. 8:**
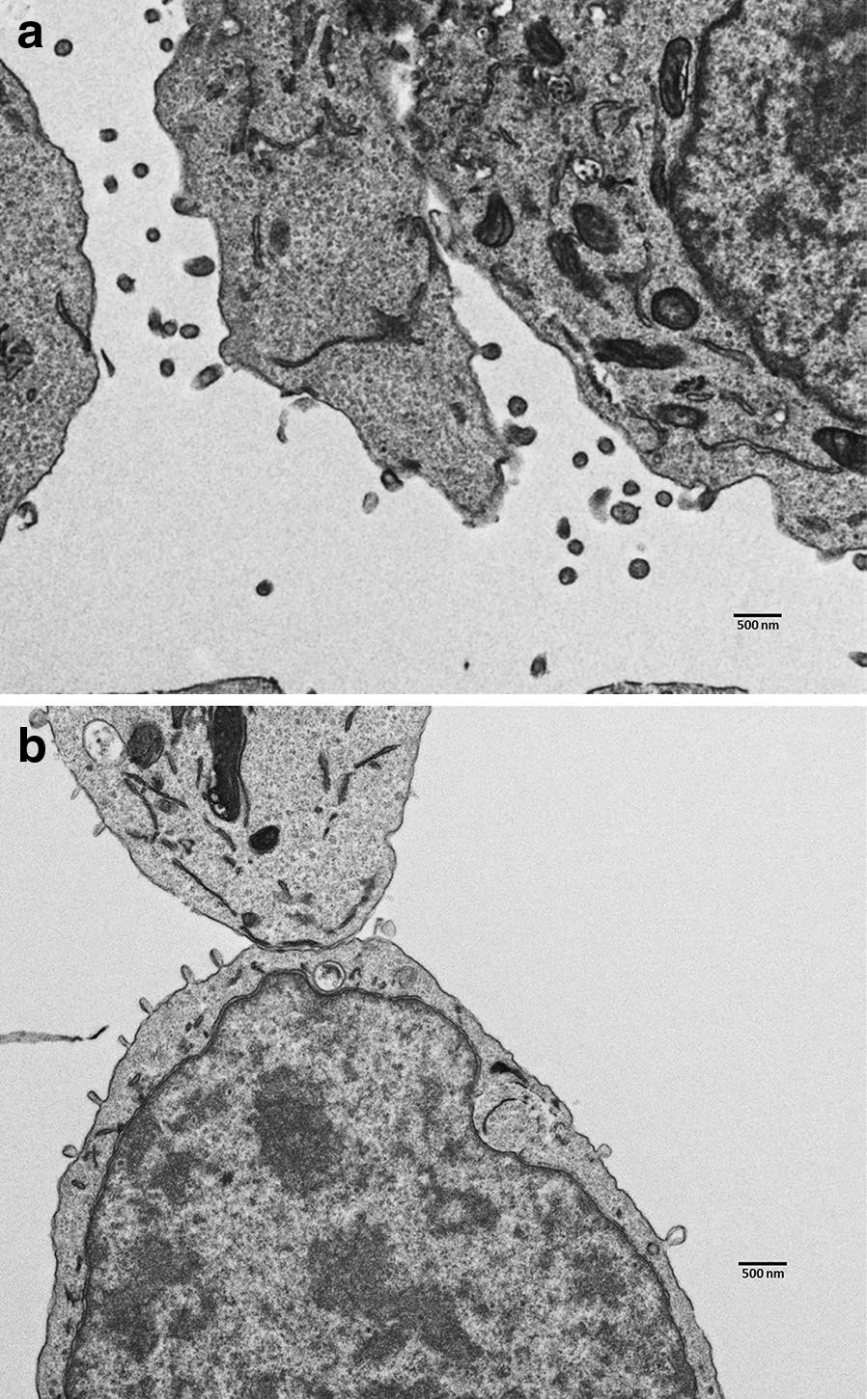
Transmission electron microscopy of thin-sectioned parental (**a**) and EAP45 knockout cells (**b**) transfected with pseudotyped HIV. Note the free virions surrounding the active budding sites in **a** and tethered budding particles on the plasma membrane in **b**. *Bar* equals 500 nm.

Defects in late domain pathways typically manifest as a failure to complete the viral budding process with significant numbers of virions arrested at an early stage of export at the plasma membrane. We examined viral budding by transmission electron microscopy in the parental and EAP45 knockout cell lines (Fig. 8). While virions can be seen budding normally from the wild type cells with free virus in the intercellular spaces, in the EAP45 knockout cells there are multiple incompletely budded virions detectable at the plasma membrane. This phenotype is very similar to that seen in other late domain defects [23–27] and provides strong evidence for the involvement of ESCRT-II in the budding process.

## Discussion

The ESCRT machinery was originally discovered in yeast where it was found to be essential for the sorting of cargos into intraluminal vesicles for degradation. Studies in yeast and in vitro reconstitution experiments showed that ESCRT-0, I, -II, -III and the Vps4-Vta1 complexes are sequentially involved in this pathway (reviewed in [28]). ESCRT-II proteins are crucial for this; overexpression of ESCRT-II rescues yeast ESCRT-I deletion mutants but not vice versa [29].

Humans possess a conserved homologous pathway and the role of ESCRT in HIV-1 budding is well established [1] with recent evidence suggesting a scaffolding interaction between the viral Gag protein and ESCRT components at the site of virus assembly [30]. The involvement of ESCRT-II has been the subject of debate for HIV and certain other systems. Specific cellular cargos including ferroportin [31] and CXCR4 [19] require ESCRT-II for their lysosomal degradation following internalisation, while others (Kaposi sarcoma-associated herpesvirus ubiquitinated MHC-I) do not [20]. For epidermal growth factor receptor (EGFR), one of the most widely studied ESCRT cargos, the data are conflicting [14, 19, 20].

Budding and release of HIV-1 is a topologically similar process to endosomal vesicle formation in which the membrane also evaginates from the cytoplasm. ESCRT-I, -III and the ESCRT-associated protein ALIX have been firmly implicated in the process. The p6 region of Gag contains two late domains, the PTAP and YPXL motifs that bind TSG101 and ALIX, respectively (reviewed in [32, 33]). Both of the TSG101 and ALIX pathways require the recruitment of certain ESCRT-III subunits and VPS4 ATPase [34, 35]. ALIX is able to recruit ESCRT-III directly [36], however in certain cell lines its contribution to HIV-1 budding is a minor one unless it is overexpressed [4, 37]. For the predominant pathway mediated by TSG101, the link between ESCRT-I and -III is still unclear. Langelier et al. concluded that HIV-1 release was ESCRT-II-independent since the depletion of the ESCRT-II subunit EAP20 by small interfering RNA did not appear to cause a significant effect on viral release and infectivity as measured up to 48 h post transfection in a pseudotyped viral system. A similar phenotype was noted using wild type virus harvested at 24 h post transfection. They did however report that, at delayed time points, knockdown of ESCRT-II had a deleterious effect on virus production [14]. Pincetic et al. showed that the fusion of EAP20 or EAP45 to the C-terminal of HIV-1 Gag containing a PTAP motif mutation did not rescue budding of virus-like particles whereas it did rescue ASLV [15]; their work also highlighted the different cell membranes involved in budding used by HIV and ASLV. It is unknown however whether the fused EAP20 and EAP45 used in their studies were properly folded and fully functional. Two copies of EAP20 are required to activate ESCRT-III [8, 12, 13] and isolated EAP45 is unstable without the other ESCRT-II partners due to its extensive exposed hydrophobic regions [7, 8, 19, 20]. How ESCRT-III is activated without ESCRT-II was unexplained [28]. Recently however a reconstituted assembly model of HIV using myristylated Gag and giant unilamellar vesicles, presented evidence that ESCRT-II was necessary for the TSG101-mediated pathway to function for viral particle assembly [16].

We have now used several established and novel approaches to study this controversial area including two different knockdown protocols with two different viral strains in different cell lines and also a CRISPR/Cas 9 EAP45 knockout cell line. In both HeLaM and 293T cell lines a defect was observed in viral export when ESCRT-II was knocked down. Importantly in no case was there a detectable decrease in cell viability at the late time points assayed, indicating that our findings were specific and did not reflect a generalised effect on vesicular sorting in the cell (Figs. 1a, 2d, Additional file 4: Figure S4C). EAP20 plays an important role in recruiting CHMP6 for correct cytokinesis [18] and the expression of ESCRT-II components is mutually dependent [19, 20 and this study]. However, the depletion of EAP45 does not noticeably affect cell proliferation suggesting the level of EAP20 is sufficient for normal cell division.

In HeLaM cells the effect of EAP45 knockdown on viral budding was masked to some extent by a decline in Gag production as measured by intracellular p24 (Fig. 1) but this decrease can now be explained as ESCRT-II appears to have an additional earlier effect on HIV protein production (Figs. 5, 6). Both knockdown and knockout experiments document a decline in intracellular Gag production indicating that there may be a transcriptional or post transcriptional effect of ESCRT-II. The magnitude of this effect seems to be cell type dependent. In 293T cells, the total p24-associated viral products are relatively unaffected (data not shown) consistent with a previous report [14]. However, in HelaM and HAP1 cells a more significant decrease in p24-associated viral products is seen (Figs. 1, 5). ESCRT-II has been shown to have transcriptional effects in other systems [38] but this has not been investigated in detail in the context of HIV. Despite the reduction in Gag production partially obscuring the later budding effect to some degree the latter is strikingly clear in the EAP45 knockout cell line (Figs. 7, 8) suggesting that ESCRT-II although not indispensable for production of infectious virus, contributes significantly to efficient particle release.

Viruses released from the PTAP mutated provirus transfected cells may be using the intact YPXL-ALIX pathway. Interestingly the fold reduction in release ratio between WT and PTAP mutant from the control cell line is similar to that from the knockout cells suggesting the proportion of viruses using YPXL-ALIX pathway is unaltered. The additive defect in infectious virus production observed after TSG101 or EAP20 are knocked down in the context of a co-transfected YPXL mutant virus, suggests that both TSG101 and EAP20 are indeed involved in the budding process (Additional file 3: Figure S3, Additional file 4: Figure S4). The time course release ratio from pulse-chase experiments corroborates this (Fig. 4). The effect seen is less dramatic but pulse chase conditions may be suboptimally sensitive to detection of alterations in viral assembly, which is very rapid [39, 40].

One of the hallmarks of the defect caused by inhibiting the ESCRT pathway is a generalised delayed rate of Gag processing which is especially marked at the final stage of cleavage of p24 from p24-p2 [2, 14, 27, 41]. We consistently see the same defect in EAP20 depleted cells (Fig. 3, Additional file 3: Figure S3) and EAP45 knock out cells (Figs. 5, 7). Similar effects in Gag processing occur in the side by side TSG101 and EAP20 knockdown conditions. The exaggerated effect of a PTAP mutant transfected into the EAP45 knock out cell line (Fig. 7d) implies that ESCRT-I and ESCRT-II may play similar or complementary roles at the early stage of virus budding. Although not cell lines for which HIV is tropic, all of these monolayer cells allow a precise assessment of a single step viral life cycle and facilitate standardisation of the infectivity assay. In addition suspension cells such as lymphocytes are less easily synchronised and cell division might be affected by ESCRT-II knockdown, which would likely impinge directly on HIV replication. The findings from gene depletion experiments (Figs. 1, 3, 4, 5, 7) together with a consistency of effect using a dominant negative interference approach (Fig. 2) strongly suggest that ESCRT-II contributes to efficient virus budding and production of infectious HIV-1 and that the effect of ESCRT-II is mediated through its interaction with ESCRT-I.

It has been suggested that the self-assembly of Gag is sufficient to deform the membrane into a bud making a role for ESCRT-II dispensable. However three dimensional analysis of the viral budding site argues that ESCRT contributes to membrane deformation during the early stage of Gag assembly and that membrane scission is not the only role of ESCRT [26]. ESCRT-II participates in membrane deformation as shown by an in vitro reconstitution assay [11] and by modelling of the crystal structure of ESCRT-II-CHMP6_2_ with membranes [12]. Our EM data confirm that in the absence of ESCRT-II components budding is slowed or arrested and help to resolve some of the controversy surrounding HIV virus release.

## Conclusion

The ESCRT system of proteins is involved in more than one process of viral production in HIV-1. ESCRT-II contributes to efficient transcriptional and/or post-transcriptional generation of HIV Gag. Additionally, there is clear evidence that the presence of ESCRT-II makes a significant contribution to the budding and viral export process of the virus and that this involves a co-ordinated link with ESCRT-I.

## Methods

### Cells

HeLaM, a derivative of HeLa cells [42] and 293T cell lines were obtained from ATCC. TZMbl, carrying two HIV-1 LTR-driven reporter genes, firefly luciferase and *E. coli* β-galactosidase [43, 44], is a HeLa cell clone that stably expresses high levels of CD4 and CCR5 and was obtained from NIH AIDS Research and Reference Reagent Program. The HAP1 EAP45 CRISPR/Cas 9 knockout and control cell lines were constructed and purchased from Haplogen GmbH, Vienna, Austria. They were grown in Iscove’s modified Dulbecco’s medium (IMDM) supplemented with 10% fetal calf serum (FCS). HeLaM, 293T and TZMbl cells were grown in Dulbecco’s modified Eagle medium (DMEM) supplemented 10% FCS. All cell lines were grown at 37°C and 5% CO_2_ incubator.

### Plasmids

pSVC21∆BglII is derived from a pHXB2 infectious clone but with a *BglII* fragment in the *env* gene (7041–7621) deleted [45, 46]. The BH10 strain was a kind gift of Michael Laughrea, McGill University, Canada. The analogous *BglII* deletion provirus was created in BH10. YPXL and PTAP deletion mutants of pBH10∆BglII were constructed using site directed mutagenesis [27, 47]. pCMV-VSV-G encodes the VSV glycoprotein for pseudotyping. pLAI is a full-length molecular clone of HIV-1 strain LAI for the expression of wild type virus [48]. pTER vector [49] was obtained from van der Wetering (Centre for Biomedical Genetics, The Netherlands) and was used for cloning shRNA into *BamHI* and *XhoI* sites to generate individual pTER-shRNA. pGEX4T1-EAP20, pGEX4T1-EAP30, pGEX4T1-EAP45 [20] and pEGFPC2-HSV1TK were obtained for the cloning of genes of interest to pEF vector containing human elongation factor 1α according to Lee et al. [50]. Mutagenesis PCR was used to introduce the IERK 159-162 AAAA mutation in the H0 helix of EAP45 [7].

### Cell viability

The manufacturer’s protocol for CellTiter-Glo Luminescent Cell Viability Assay (Promega) was adapted for 96-well half-area plates. Briefly, the cell culture plate was equilibrated at room temperature for 30 min. CellTiter-Glo Reagent (50 µl) was added to each well and the plate was placed on an orbital shaker (750 rpm, Titramax 100, Heidolph) for 2 min to lyse cells. After a further 10-min incubation at room temperature, luminescence was read with a 1 s integration time using GloMax 96 Microplate Luminometer (Promega).

### Pseudotyped and wild type virus production with shRNA or EAP45 expression

To produce pseudotyped virus, HeLaM cells of 50–80% confluency were co-transfected in a 24-well plate format with 44 ng pSVC21ΔBglII and 15.6 ng pCMV-VSV-G in an optimised ratio of 3.7:1.3 using Fugene HD (Roche). A total amount of 200 ng pBH10∆BglII and pCMV-VSV-G was co-transfected into 50–80% HAP1 cells in a 24-well plate using TurboFectin 8.0 (Origene). For wild type virus, cells were transfected with 40 ng pLAI. Transient expression of shRNA was performed by co-transfecting pTER-shRNA with the viral plasmids. For pseudotyped virus, 120 ng shRNA expression plasmid together with the above mentioned quantities of DNA plasmids for pseudotyping were transfected. For wild type virus, 50 ng shRNA expression plasmid and 40 ng LAI were co-transfected. In the EAP45 mutant experiments HeLaM were transiently co-transfected with 200 ng EAP45 mutant expression plasmids. 200 ng EAP45 mutant expression plasmids was also used for the wild type virus study. In all the above experiments, viruses were harvested at 48 or 96 h post-transfection and the supernatant was used to infect TZMbl cells to assay for infectivity. The medium was replaced 24 h prior to harvesting at 96 h to study the released viruses at late point. Supernatant was inactivated in Empigen (0.1%; Sigma-Aldrich) in PBS. Virus-producing cells were lysed in 150 μl/well of 1% Empigen in Passive Lysis Buffer (Promega). Extracellular and intracellular CA-p24 levels were quantified by ELISA (Aalto) with a slight modification [51].

### Virus infectivity

Infectivity was based on the Tat-dependent upregulation of LTR-driven firefly luciferase expression upon HIV-1 infection of TZMbl cells. Cells (~10^5^) were seeded in 24-well plates in 0.5 ml/well of complete DMEM. Equal volumes of medium from virus-producing cells were added to TZMbl cells. DEAE-dextran (50 µg/ml; AppliChem) was added to facilitate infection. TZMbl cells were lysed at 48 h post-infection in 150 µl/well of Cell Culture Lysis Reagent (CCLR; Promega). Firefly luciferase expression was quantified using the Luciferase Assay System (Promega). Cell lysates (5 µl) were transferred to a white 96-well half-area plate. Luciferase Assay Reagent (25 µl) was added and the luminescence was measured by GloMax 96 Microplate Luminometer (Promega).

### RNA FISH

Around 1 × 10^5^ either HAP1 control or EAP45 KO cells were seeded in each well of an 8 well multi-well glass slide (Millipore). After 24 h, the cells were infected with HIV-VSV-G containing 200 ng supernatant p24. After 24 h infection, the media was removed and washed once with PBS before fixing with formaldehyde in PBS (3.7%) for 10 min at room temperature. This was followed by washing with PBS and permeabilising with 70% ethanol at 4°C for 1 h. The probe set composing 37 oligonucleotides labelled with Quasar^®^ 570 Dye at the 5′ end was purchased from Biosearch Technologies specifically targeting HIV Gag coding region. The hybridisation process was followed according to manufacturer’s instructions. The vectashield was applied before the coverslip was mounted and visualised under Olympus Ix81 fluorescence microscope. The TRITC channel was used for genomic RNA visualisation. The images were taken by pre-setting at 300 ms exposure time. For fluorescence quantitation, the genomic RNA positive cells were identified and the area of each individual cell was defined and quantified using ImageJ (NIH).

### siRNA knockdown

The Stealth siRNA (Life technologies) knockdown was performed using TransIT-TKO transfection reagent (Mirus) based on the manufacturer’s instructions. Briefly, one well of 50% confluent 293T cells in a 24 well plate was transfected with 2 µl, 20 µM Stealth siRNA to EAP20, TSG101 or control using 2.5 µl TransIT-TKO. Total amount of 40 ng pBH10∆BglII and pCMV-VSV-G was mixed with 160 ng pBluescript empty vector and followed by co-transfection with second dose of 2 µl, 20 µM siRNA 24 h post first dose transfection. The supernatant was collected 72 h post first dose transfection and clarified by low centrifugation before ELISA for p24 quantitation or Optiprep cushion ultracentrifugation for virion purification for Western analysis. Total cells were lysed with 150 µl 1× CCLR before being subjected to ELISA and Western blot analysis using mouse monoclonal antibodies to TSG101 (Abcam) and to HIV-1 p55/p24 (ARP313, NIBSC) and rabbit polyclonal antibodies to GAPDH (Abcam) and EAP20 (P Luzio, Cambridge Institute for Medical Research, UK). Quantification of Western blots was performed using ImageJ (NIH). For knockdown conditions for the pulse-chase experiments one well of fifty percent confluent 293T cells in a 6 well plate was transfected with 8 µl, 20 µM Stealth siRNA to EAP20, TSG101 or control using 10 µl TransIT-TKO. A total of 2 µg pBH10∆BglII and 135 ng pCMV-VSV-G was co-transfected with second dose of Stealth siRNA as above. After 48 h post first transfection, the cells were labelled with ^35^S protein labeling mix (Perkin-Elmer) as described in the pulse-chase method section.

### Pulse-chase analysis

Pulse-chase analysis was performed by adapting the method of L’Hernault et al. [52]. Briefly, 293T cells were transfected with 2 µg pBH10∆BglII and 135 ng pCMV-VSV-G in 6-well plates. After 24 h, cells were washed and starved in 0.5 ml of DMEM lacking methionine and cysteine (Met-Cys-free DMEM; Sigma-Aldrich) supplemented with 4 mM glutamine and 10% FCS. Cells were then pulse-labelled for 30 min with 0.5 ml of Met-Cys-free DMEM supplemented with 110 μCi of EasyTag Express ^35^S protein labeling mix (11 mCi/ml; Perkin-Elmer). The medium was removed and supplemented with fresh complete DMEM with 2 mM Met and 2 mM Cys when time point zero was set. Cells were chased at 90 and 180 min post labelling. At each time point the virus-containing supernatants were harvested and clarified by low speed centrifugation. Cells were lysed with RIPA buffer (50 mM Tris–HCl [pH 7.5], 100 mM NaCl, 1% sodium deoxycholate, 0.1% SDS, 1% Triton X-100, protease inhibitor cocktail [Roche]). The capsid and Gag proteins were immunoprecipitated using 3.25 µg anti-HIV-1 p55/p24 mouse monoclonal antibody (ARP313, NIBSC) at 4°C. Protein G-Sepharose beads (Sigma-Aldrich) were then added to the mixtures for a further 1 h rotation before being spun and washed in RIPA buffer. Finally, the beads were resuspended in 50 μl 2× Laemmli buffer. Samples were boiled for 5 min before being loaded onto a 15% SDS-polyacrylamide gel. Gels were fixed and dried prior to autoradiography. Quantification was performed using ImageJ (NIH).

### Electron microscopy of budding virus

The HAP1 control cells and KO cells were transfected as described above. 48 h post transfection, the supernatant was removed and the monolayer of cells was washed with 9% NaCl followed by fixation in 2% glutaraldehyde and 2% formaldehyde in 0.05 M cacodylate buffer overnight. The cells were scraped and collected by centrifugation before being embedded and thin-sectioned. The thin-sectioned samples were mounted on an EM grid and visualised using a Tecnai G2 electron microscope. Images were taken at 3,500× magnification.

## Additional files

Additional file 1:
**Figure S1.** Validation of shRNA system for the study of HIV-1 replication. (A) Western blot detecting the knockdown of DDX3 by shDDX3 (target sequence CATTGAGCTTACTCGTTAT) in HeLaM cells. (B) Cell viability assay performed as described for Figure 1A. (C-D) Production of infectious pseudotyped (c, mean ± SEM, n = 4) and wild type (d, mean ± SD of duplicate samples) viruses upon knockdown of DDX3, 96 h post-transfection. Transfections and analyses were performed as described for Figure 1. Additional file 2:
**Figure S2.** Inhibition of pseudotyped virus production by knockdown of ESCRT-II. As for Figure 1 (A: EAP45; B: EAP20; C: EAP30) showing results at 48 h post transfection. Additional file 3:
**Figure S3.** Budding effects from YPXL^−^ transfected cells upon knocking down TSG101 or EAP20. (A) as described in Figure 3A except YPXL mutant provirus was used in the transfection. (B) The supernatant extracellular p24 from YPXL^−^ transfected cells under each condition was normalised against to that of WT siControl from Figure 3. (D) Extracellular p24 is divided by intracellular p24 to give the release ratio before being normalised to WT siControl from Figure 3. Error bars represent the SEM from six replicates. Additional file 4:
**Figure S4.** Characterisation of CRISPR/Cas9 EAP45 knockout HAP1 cell line. (A). Sanger sequencing confirms that there is a 20 nt deletion (underlined) in exon 3 of HAP1 EAP45 knockout cell line. (B). The transfection efficiency of both control and knockout cell lines were monitored by transfecting a eGFP expression construct. (C). Cell viability assay of WT provirus transfected HAP1 and KO cells. Non-transfected and blasticidin treated cells served as controls. The error bars represent the SEM from two replicates.
