# Supplementary figures and images for: Evidence that the endosomal sorting complex required for transport-II (ESCRT-II) is required for efficient human immunodeficiency virus-1 (HIV-1) production
Source: Retrovirology. 2015 Aug 14;12:72. doi: 10.1186/s12977-015-0197-x (PMC4535389; doi:10.1186/s12977-015-0197-x)

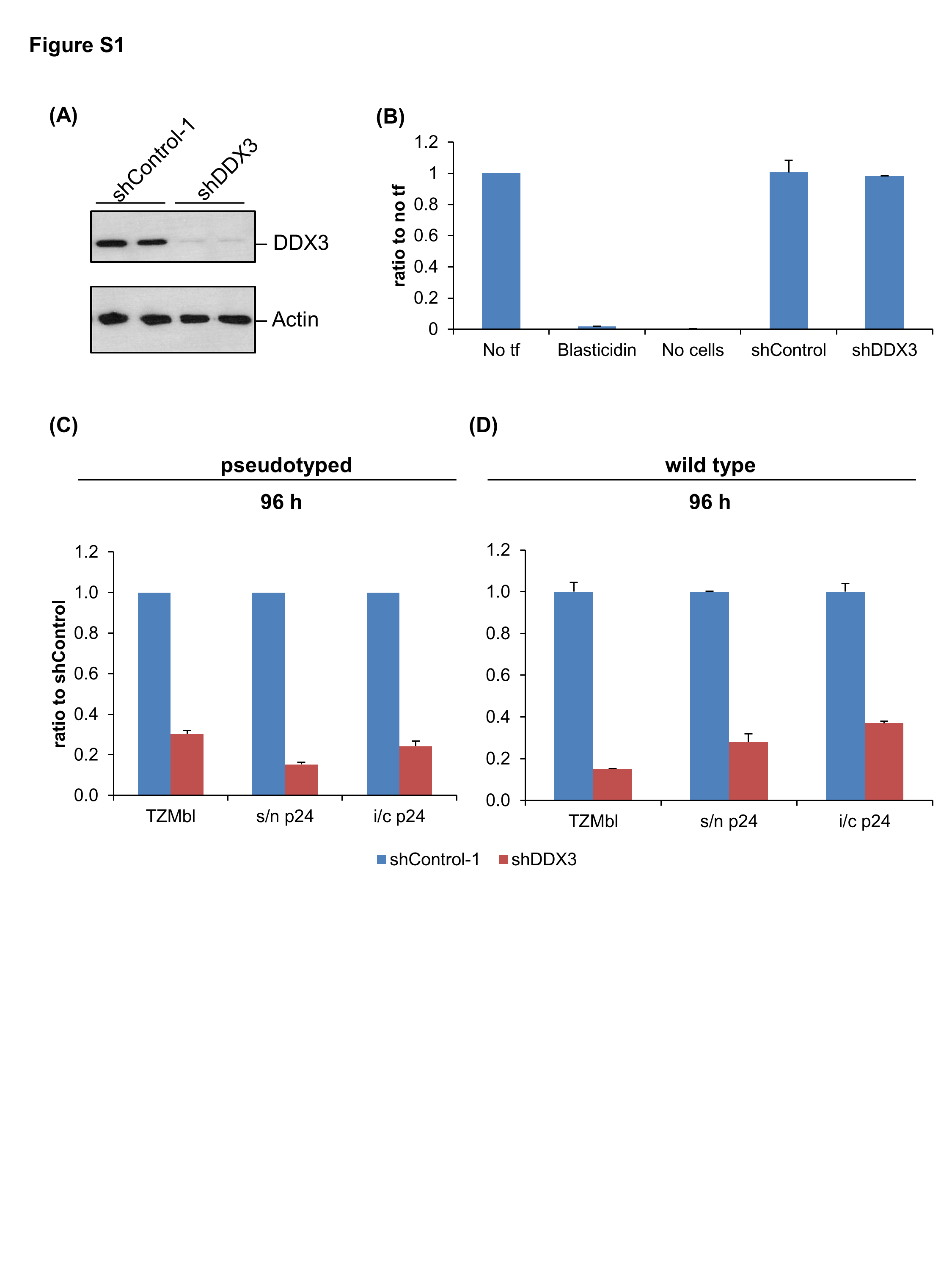

Supplement: Additional file 1: — Figure S1. Validation of shRNA system for the study of HIV-1 replication. (A) Western blot detecting the knockdown of DDX3 by shDDX3 (target sequence CATTGAGCTTACTCGTTAT) in HeLaM cells. (B) Cell viability assay performed as described for Figure 1A. (C-D) Production of infectious pseudotyped (c, mean ± SEM, n = 4) and wild type (d, mean ± SD of duplicate samples) viruses upon knockdown of DDX3, 96 h post-transfection. Transfections and analyses were performed as described for Figure 1. [file 12977_2015_197_MOESM1_ESM.tif]

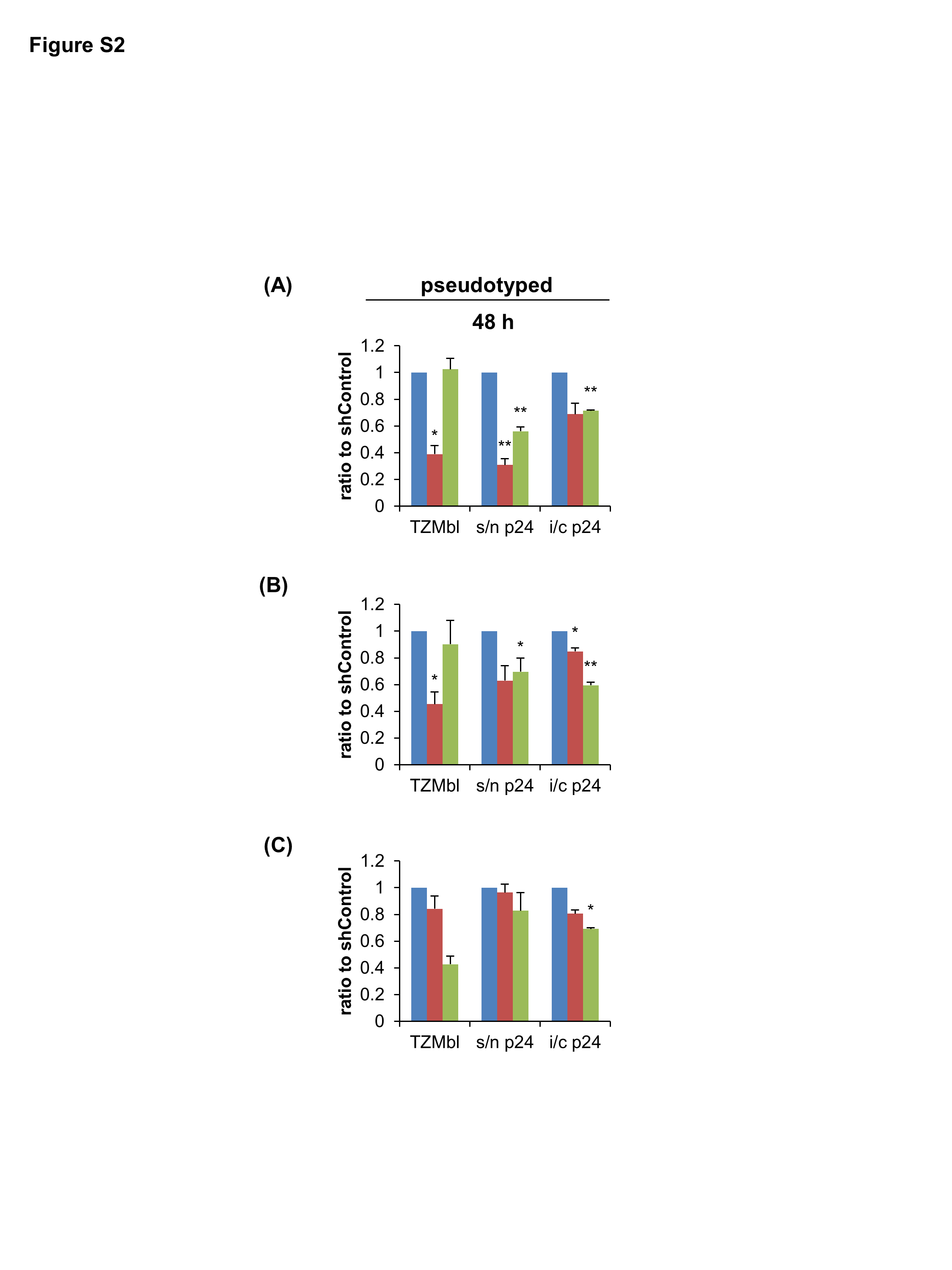

Supplement: Additional file 2: — Figure S2. Inhibition of pseudotyped virus production by knockdown of ESCRT-II. As for Figure 1 (A: EAP45; B: EAP20; C: EAP30) showing results at 48 h post transfection. [file 12977_2015_197_MOESM2_ESM.tif]

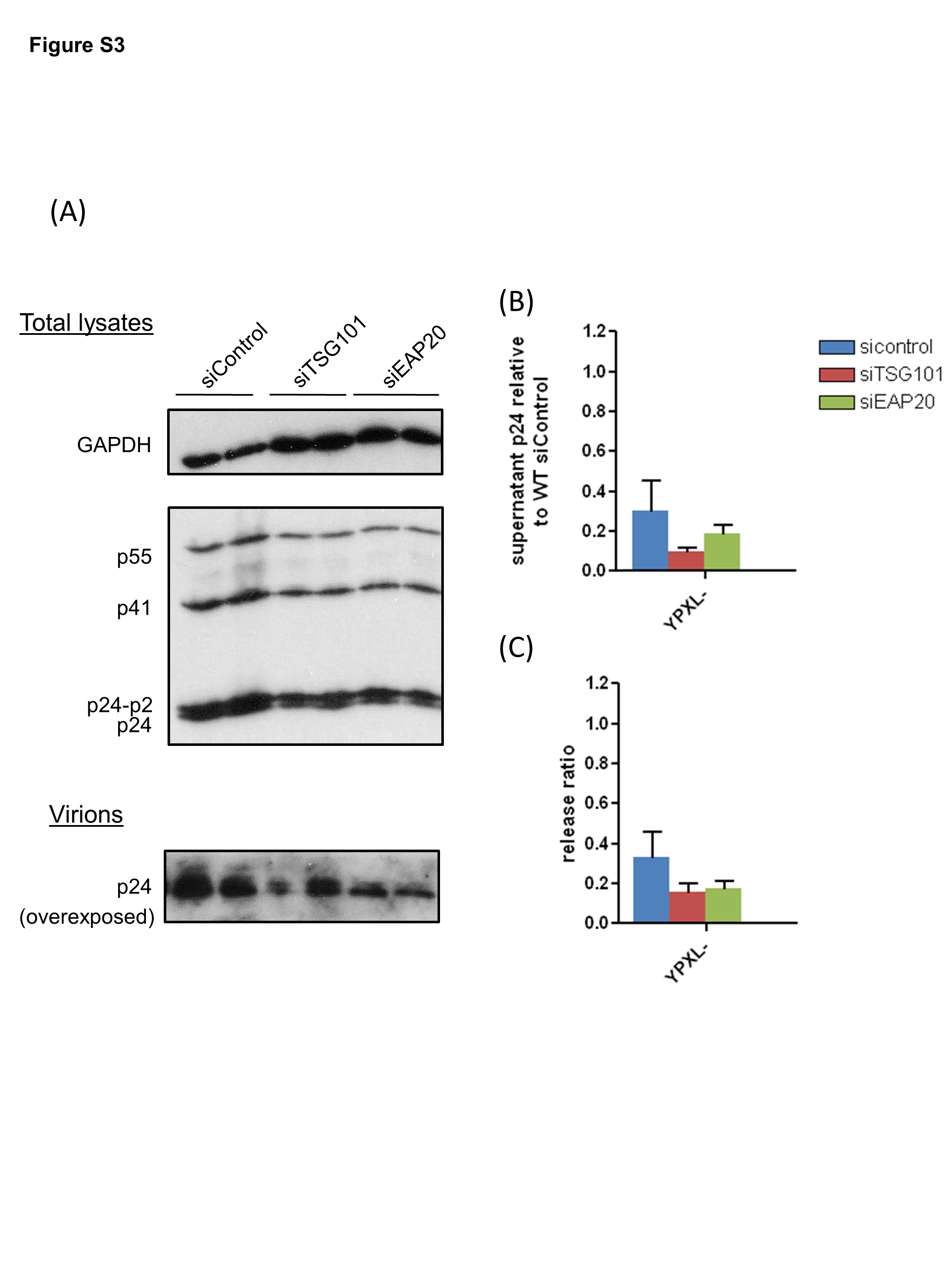

Supplement: Additional file 3: — Figure S3. Budding effects from YPXL− transfected cells upon knocking down TSG101 or EAP20. (A) as described in Figure 3A except YPXL mutant provirus was used in the transfection. (B) The supernatant extracellular p24 from YPXL− transfected cells under each condition was normalised against to that of WT siControl from Figure 3. (D) Extracellular p24 is divided by intracellular p24 to give the release ratio before being normalised to WT siControl from Figure 3. Error bars represent the SEM from six replicates. [file 12977_2015_197_MOESM3_ESM.tif]

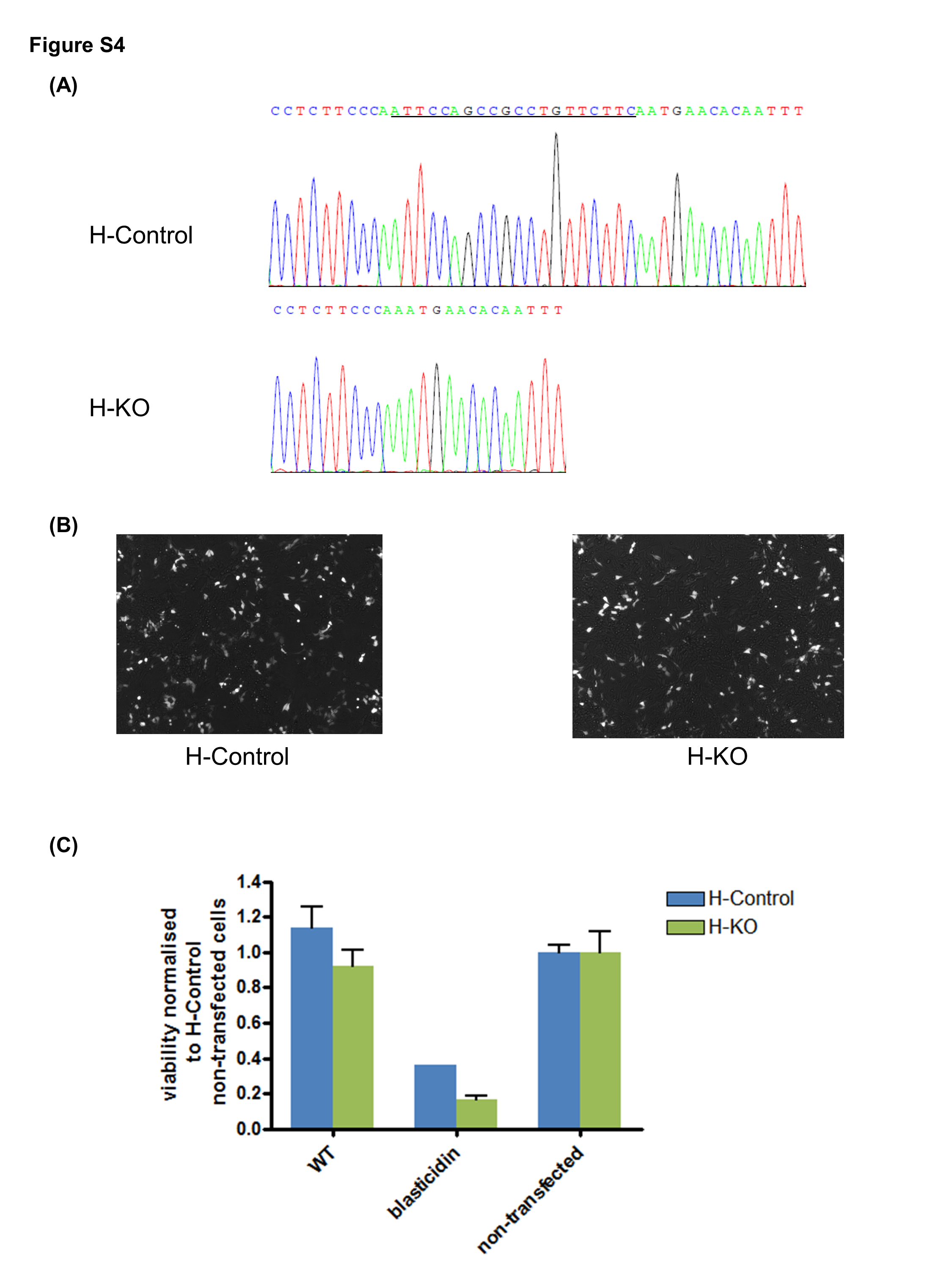

Supplement: Additional file 4: — Figure S4. Characterisation of CRISPR/Cas9 EAP45 knockout HAP1 cell line. (A). Sanger sequencing confirms that there is a 20 nt deletion (underlined) in exon 3 of HAP1 EAP45 knockout cell line. (B). The transfection efficiency of both control and knockout cell lines were monitored by transfecting a eGFP expression construct. (C). Cell viability assay of WT provirus transfected HAP1 and KO cells. Non-transfected and blasticidin treated cells served as controls. The error bars represent the SEM from two replicates. [file 12977_2015_197_MOESM4_ESM.tif]
